# Innovative Application of Diathermy in Orthodontics: A Case Report

**DOI:** 10.3390/ijerph19127448

**Published:** 2022-06-17

**Authors:** Nunzio Cirulli, Alessio Danilo Inchingolo, Assunta Patano, Sabino Ceci, Grazia Marinelli, Giuseppina Malcangi, Giovanni Coloccia, Valentina Montenegro, Chiara Di Pede, Anna Maria Ciocia, Giuseppe Barile, Antonio Mancini, Giulia Palmieri, Daniela Azzollini, Biagio Rapone, Ludovica Nucci, Ioana Roxana Bordea, Antonio Scarano, Felice Lorusso, Gianluca Martino Tartaglia, Cinzia Maspero, Manuel Nuzzolese, Filippo Cardarelli, Daniela Di Venere, Angelo Michele Inchingolo, Gianna Dipalma, Francesco Inchingolo

**Affiliations:** 1 Department of Interdisciplinary Medicine, University of Bari “Aldo Moro”, 70124 Bari, Italy; dottore@studiocirulli.it (N.C.); ad.inchingolo@libero.it (A.D.I.); assuntapatano@gmail.com (A.P.); s.ceci@studenti.uniba.it (S.C.); graziamarinelli@live.it (G.M.); giuseppinamalcangi@libero.it (G.M.); giovanni.coloccia@gmail.com (G.C.); valentinamontenegro@libero.it (V.M.); c.dipede1@studenti.uniba.it (C.D.P.); anna.ciocia1@gmail.com (A.M.C.); g.barile93@hotmail.it (G.B.); dr.antonio.mancini@gmail.com (A.M.); giuliapalmieri13@gmail.com (G.P.); daniela.azzollini93@gmail.com (D.A.); biagiorapone79@gmail.com (B.R.); drfilippocardarelli@libero.it (F.C.); daniela.divenere@uniba.it (D.D.V.); angeloinchingolo@gmail.com (A.M.I.); giannadipalma@tiscali.it (G.D.); francesco.inchingolo@uniba.it (F.I.); 2 Multidisciplinary Department of Medical-Surgical and Dental Specialties, University of Campania Luigi Vanvitelli, Via L. De Crecchio 6, 80138 Naples, Italy; ludovica.nucci@unicampania.it; 3 Department of Oral Rehabilitation, Faculty of Dentistry, Iuliu Hațieganu University of Medicine and Pharmacy, 400012 Cluj-Napoca, Romania; 4 Department of Innovative Technologies in Medicine and Dentistry, University of Chieti-Pescara, 66100 Chieti, Italy; ascarano@unich.it; 5 UOC Maxillo-Facial Surgery and Dentistry, Department of Biomedical, Surgical and Dental Sciences, School of Dentistry, Fondazione IRCCS Ca Granda, Ospedale Maggiore Policlinico, University of Milan, 20100 Milan, Italy; gianluca.tartaglia@unimi.it (G.M.T.); cinzia.maspero@unimi.it (C.M.); 6 Nottingham University Hospitals, Nottingham NG5 1PB, UK; manuzzolese@hotmail.com

**Keywords:** dentistry, orthodontics, orthodontic brackets, tooth movement techniques, diathermy, electrotherapy

## Abstract

Introduction: Several strategies have been proposed in the literature to accelerate tooth movement, many of which are invasive and have numerous side effects, such as surgical techniques (corticotomy and piezocision technique). This research investigates to what extent diathermy can accelerate the orthodontic alignment phase. Materials and Methods: A patient with lower teeth crowding index of the same magnitude was selected. The orthodontic treatment with Nickel–Titanium (NiTi) thermal arc 0.015 in the lower arch was performed, associated with a weekly application of diathermy using the intraoral handpiece. The total duration of treatment was three weeks. During each session, an intraoral transducer was employed to stimulate the hard and soft tissues of the left dental hemiarch, which was also orthodontically aligned like the right one. Results: Comparing the tooth movements of four elements of the two hemiarchies, it was found that, overall, the two teeth examined on the treated side underwent a more significant number of changes than on the untreated side, although not by a significant amount. Conclusions: The use of diathermy, according to the authors, is a non-invasive approach that may speed up the orthodontic alignment phase and reduce treatment duration, resulting in a lower risk of caries, gingival recessions, root resorptions, and patient compliance improvement, without side effects. Further studies and an adequate sample size will be needed to confirm the findings.

## 1. Introduction

Over the years, there has been a tendency to reduce orthodontic treatment time to minimise the onset of caries, gingival recession, and root resorption, but above all, to meet patients’ demands. Numerous studies have been proposed in the literature regarding the acceleration of orthodontic movement with minimal adverse effects. Transeptal fibertomy, a surgical procedure characterised by the detachment of marginal gingiva from the root surfaces, has improved the speed of movement of teeth and reduced relapses during orthodontic therapy in a rat model [1]. In a three-month evaluation trial, low-level laser therapy using a gallium-aluminium-arsenide (GaAlAs) diode had no effect [2]. A systematic review by Bartzela et al. [3] evidenced the influence of medications and diary on orthodontic tooth movement, especially in animal models: parathyroid hormone (PTH), eicosanoids, corticosteroid hormones, thyroxin, and vitamin D3 stimulates tooth movement. However, from a systematic review conducted by Khurshid [4], it is impossible to state that PTH increases tooth displacement speed during orthodontic treatment. In fact, the animal studies analysed contained numerous biases and were not long term.

In the twentieth century, Sandstedt described the impact of mechanical force on the periodontal ligament–alveolar bone interface. His work clearly illustrated that the teeth movement caused by orthodontic forces is due to the alveolar bone resorption on the pressure side. In contrast, bone apposition occurs on the tension side [5,6,7]. The movement of these elements is related to the magnitude force and the biological tissue responses. The force applied causes alterations in the microenvironment around the PDL, such as changes in blood flow and secretion of various inflammatory mediators (cytokines, colony-stimulating growth factors, and arachidonic acid metabolites). As a result, bone remodelling occurs [8,9,10]. In the literature, the numerous scientific controversies concerning the acceleration of the orthodontic movement all have a minimum common denominator: “How is it possible to speed up dental movements”? Several therapeutic approaches have been proposed: a biological approach that uses molecules, such as prostaglandin E (PGE), cytokines, lymphocytes, monocyte-derived factors, receptor activator of nuclear factor-kappa-Β ligand B (RANK-L), and macrophage colony-stimulating factor (MCSF), or a surgical approach, such as alveolar interseptal surgery, osteotomy, corticotomy, and piezocision technique [8,11]. Another method proposed to speed up orthodontic treatment is diathermy, an endogenous thermotherapy that uses electric currents, induced by a monopolar capacitive/resistive radiofrequency at 448 kHz, to generate deep tissue heating. Its use in clinical practice has been relatively common for nearly 20 years, but clinical efficacy has only recently been evaluated. Most studies have reported encouraging results in pain relief and functional improvement in various musculoskeletal conditions [12,13,14,15]. Diathermy can support the healing processes of injured/dysfunctional tissues thanks to its thermo-therapeutic effect and its ability to influence blood flow. However, can it affect blood flow in the most superficial layers? A diathermy device provides two different treatment modes: capacitive (CAP) and resistive (RES). These modes are usually provided by various probes (electrodes) made of medical stainless steel. The two treatment options induce different tissue reactions depending on the strength of the target tissue. Only heat is generated in the superficial layers when the active electrode is provided with an insulating ceramic layer that acts as a dielectric medium (CAP). There is a selective action on low-impedance soft tissues (rich in water), such as fatty tissue, muscle, cartilage, and lymphatic tissue. If the active electrode is not provided with an insulating layer (RES), the radiofrequency energy passes directly through the body toward the inactive electrode. This generates heat in the most resistant (low water content) and deepest tissue layers, i.e., bones, muscle groups, and tendons, causing the acceleration of the orthodontic movement [16,17,18,19]. The study aims to evaluate the use of diathermy technology in orthodontic patients as an adjuvant in tooth movement.

## 2. Materials and Methods

### 2.1. Inclusion and Exclusion Criteria

The patient was selected with the following inclusion criteria:

- Marked bilateral mandibular dental crowding of equal entity;
- Presence of all dental elements;
- Good hygienic conditions;
- Between 11 and 25 years of age.

Exclusion criteria:

- Bilateral uneven right- and left-sided tooth crowding;
- Developmental age.

### 2.2. Case Description and Treatment Protocol

A 24-year-old Caucasian male was the participant. During the first evaluation, the personal and medical history of the patient was collected. Subsequently, the necessary orthodontic documentation was obtained: dental impressions, intra- and extra-oral photographs, orthopantomography X-ray (OPG), and lateral–lateral teleradiography X-ray of the skull. A molar and canine Angle class I malocclusion with mandibular dental crowding was diagnosed at the clinical examination.

**Figure 1:**
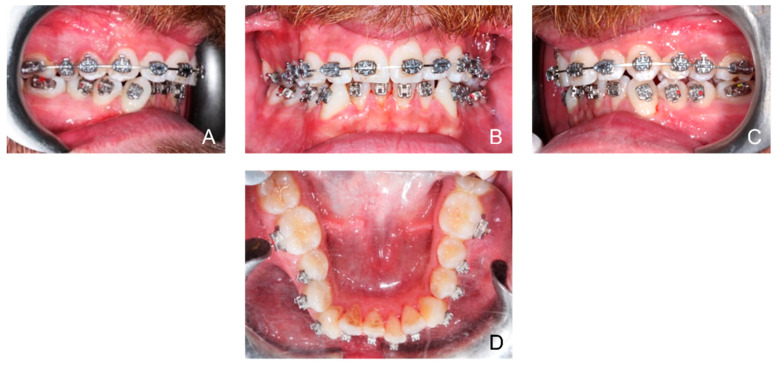
Initial patient intraoral photos: right hemiarcs view (**A**); front view (**B**); left hemiarcs view (**C**); occlusal lower arch (**D**).

**Figure 2:**
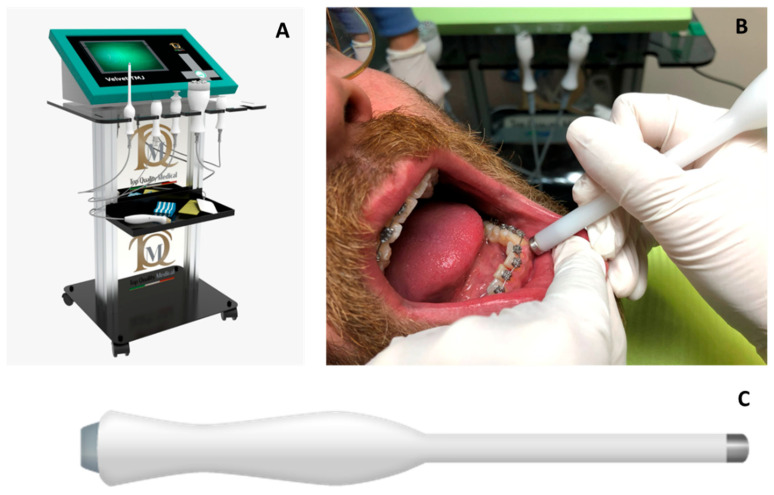
Photo of the Velvet TMJ (Top Quality Medical) (**A**); clinical application of the intraoral handpiece (**B**); stainless steel intraoral handpiece (**C**).

**Figure 3:**
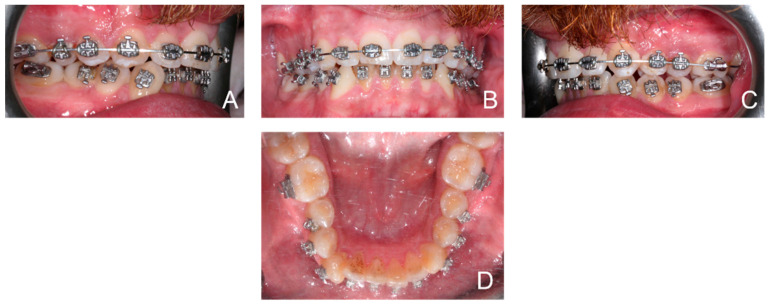
Patient intraoral photos three weeks after therapy started: right hemiarcs view (**A**); front view (**B**); left hemiarcs view (**C**); occlusal lower arch (**D**).

**Figure 4:**
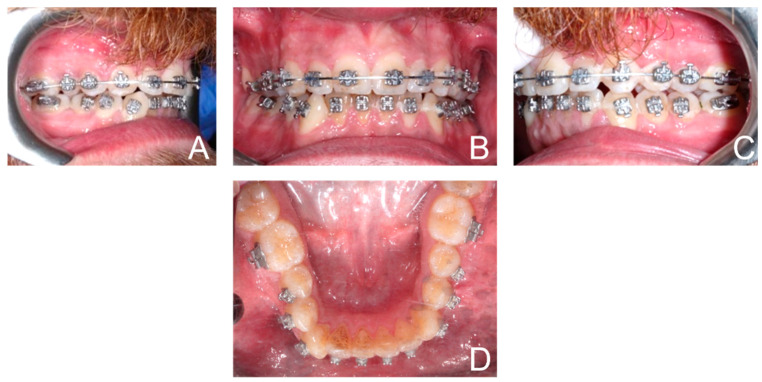
Final patient intraoral photos: right hemiarcs view (**A**); front view (**B**); left hemiarcs view (**C**); occlusal lower arch (**D**).

**Figure 5:**
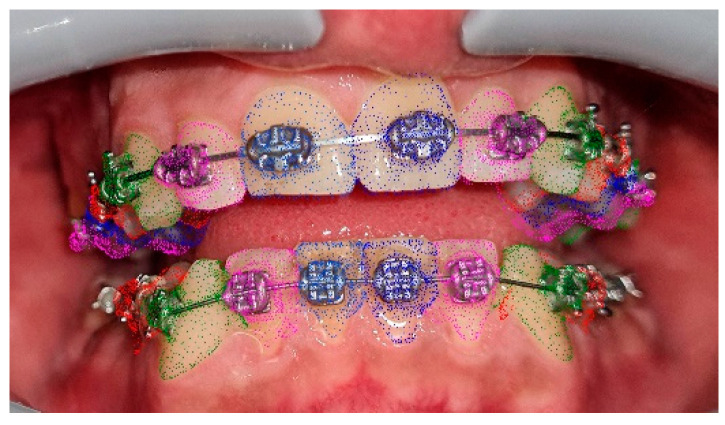
Anatomical findings evaluated by DentalMonitoring software (Dental Monitoring SAS, Paris, France). Each colour identifies the movement of a precise tooth.

Orthodontic multibrackets treatment was performed to realign teeth and improve the bite. The patient underwent banding with lower synergy brackets and a 0.015-inch NiTi thermal arch (Figure 1). Immediately after banding, the first application of diathermy was performed. A metal plate was placed in the back region to direct the radiowaves, generated by the handpiece, at a controlled frequency and direction. A conductive gel was used between the metal plate and the back skin to reduce the impedance. Intraorally, a gel was applied to the area of interest to improve the massage. Subsequently, the intraoral handpiece of the Velvet TMJ (Top Quality Medical) equipment was used between the marginal gingiva and the mucogingival line to stimulate only the patient’s left lower hemiarch during each session, leaving the contralateral hemiarch as a control (Figure 2). Each single-polar resistive session (1000 kHz) lasted 6 min at 7% power in “fracture consolidation treatment” mode. Diathermy stimulation was performed each week on the same side for three weeks. The total duration of treatment with diathermy was three weeks, and after this period, intraoral photos were taken (Figure 3). Considering that the first five weeks are essential for dental alignment, new intraoral photos were taken after seven weeks (Figure 4), and the alignment obtained on the treated side compared to the control side was evaluated. Tooth movement was measured and quantified through an artificial-intelligence-based system, DentalMonitoring (by Dental Monitoring) (Figure 5). The latter guaranteed the authors a perfect control of dental movements after orthodontic treatment. There are two articles in the literature highlighting the effectiveness of this software in monitoring orthodontic movements during treatments with the rapid palatal expander [20,21]. Immediately after the first diathermy application, the patient took the first intraoral photo, uploading it directly to the application. The last photo was taken after one month of treatment, and a comparison was made between the position of the treated half-arch and the control one.

**Figure 6:**
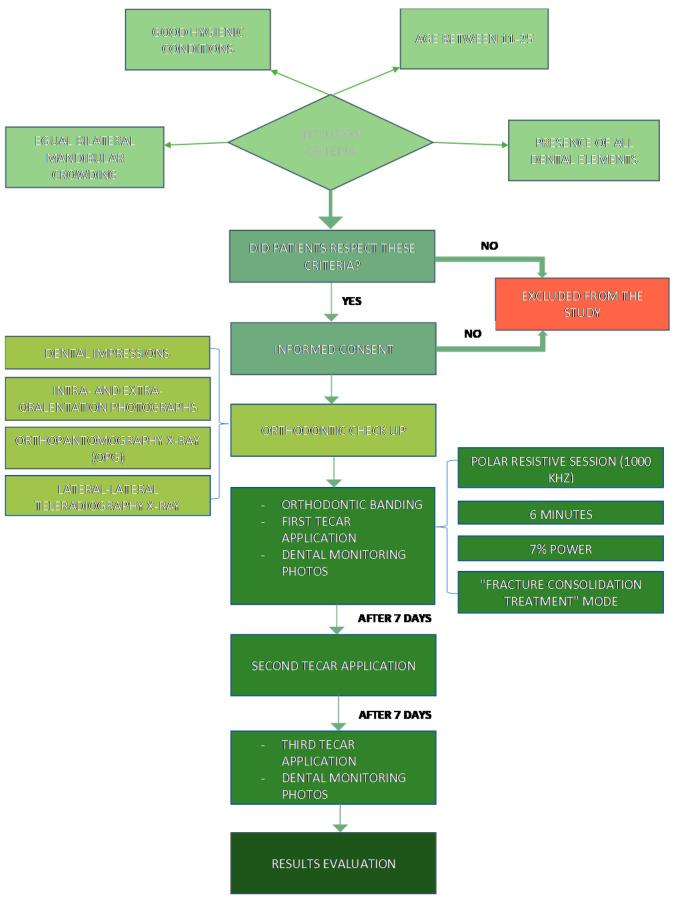
Materials and Methods flow chart.

A flow chart summarising the Materials and Methods is reported in Figure 6 to make the patient selection processes and therapeutic procedures adopted clearer.

## 3. Results

**Figure 7:**
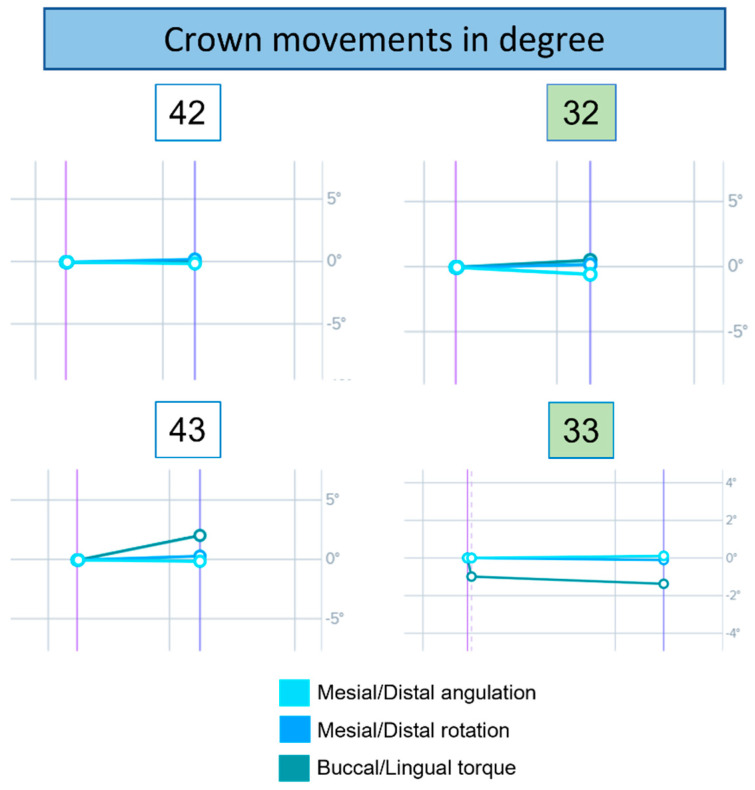
Graphs of the crown movement of elements 32, 33, 42, and 43. Each line corresponds to the movement along an axis and is colour coded.

**Figure 8:**
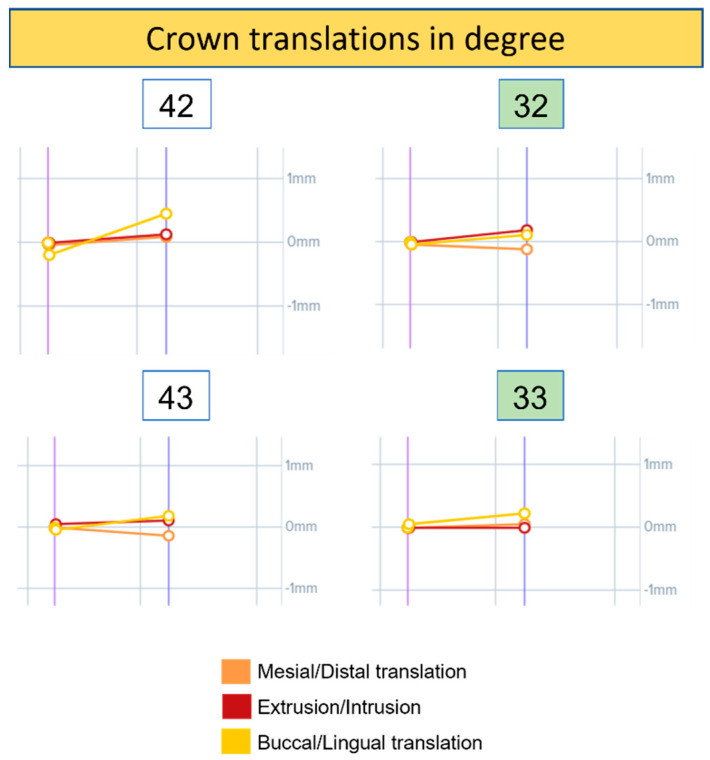
Graphs of the crown translation of elements 32, 33, 42, and 43. Each line corresponds to movement along an axis and is colour coded.

The changes in crown movement and translation at t0 and t1 are shown in the DentalMonitoring^®^ software (Dental Monitoring SAS, Paris, France) graphs in Figure 7 and Figure 8. The movement of each element was assessed in three planes of rotation and three planes of translation with unique colour segments for the two graph types. Results obtained over a 4-week evaluation were:

- 32 showed a slight variation in angulation and torque compared to 42. The rotation is unchanged in both cases.
- 33 and 43 reached the same absolute torque value at the end of the observation period. However, 43 had a greater variation. Angulation and mesiodistal rotation were almost unchanged in both cases.
- 32 showed a greater variation in mesiodistal translation and intrusion–extrusion than element 42. The latter, however, had a little variation in buccolingual translation.
- 33 showed a greater variation in buccolingual translation compared to 43. The latter had a greater variation in mesiodistal translation than 33.

## 4. Discussion

In the literature, to speed up dental movement, surgical techniques, such as alveolar interseptal surgery, osteotomy, corticotomy, and the piezocision technique, are considered safe and effective in reducing orthodontic treatment duration [22,23,24,25]. The limitations of these techniques are: formation of face and subcutaneous neck hematomas following intensive corticotomies and acceleration in the first 3–4 months only [8,12,16,22,26]. Doctors and patients have always preferred non-surgical approaches as they are non-invasive and comfortable. These techniques range from administering biological molecules to innovative physical stimulation technologies [27,28,29]. In particular, prostaglandins exogenous application influences bone remodelling and accelerates tooth movement; however, these agents increase the risk of root resorption and pain [30,31,32,33,34]. Other studies have shown how the administration of other agents, such as epidermal growth factor (EGF), parathyroid hormone (PTH), 1.25 dihydroxyvitamin D3, and osteocalcin, accelerate the effects; however, their safety and efficacy in humans need to be further proven [35,36,37,38,39,40]. Recent works have shown that through the High-Frequency Acceleration (HFA) physical stimulation technique, the vibration at a given frequency produces a significant localised catabolic effect, so strong as to increase the speed of movement of the teeth in response to orthodontic forces. Furthermore, the different vibration characteristics can significantly affect the speed of tooth movement [41,42,43,44]. Among other physical stimulation techniques, Osti R. describes TECAR as an endogenous thermotherapy (diathermy) that uses electric currents, generating deep tissue heating. The device consists of a short-wave current generator and electrodes with multi-frequency emission systems [45,46,47,48,49]. Radiofrequency is used in clinical practice to relieve pain and inflammation and improve tissue extensibility in various musculoskeletal conditions, such as low-back pain, tendinopathies, etc. Its efficacy in influencing blood flow, consequently to its thermo-therapeutic effect, is considered one of the many ways diathermy supports the healing processes of injured tissues [12,16,50,51,52]. The mechanism of action of diathermy involves an increase in vascularisation with a consequent increase in tissue oxygenation, leading to cellular stimulation. Most cellular activity can be accelerated and/or increased [12,53,54]. This study is based on the hypothesis that diathermy promotion of cell proliferation and differentiation may favour an acceleration in orthodontic movements. In the literature, no studies highlight the acceleration of the orthodontic movement with radiofrequency.

“DentalMonitoring” was used to evaluate dental movement. It consists of a mobile application that uses a patented algorithm to track movements. It also presents a doctor dashboard that updates the clinician on the movement evolution. The device effectiveness was evaluated by Impellizzeri et al. [55].

This study shows that radiofrequency could effectively accelerate orthodontic movement, even with small margins. The limitations of this work are the absence of literature comparison, the short monitoring period, and the study type. Furthermore, it should be considered that individual characteristics, including age, gender, bone metabolism, oral environment, and periodontal status, all influence orthodontic tooth movement in different people [56].

## 5. Conclusions

In this work, diathermy has suggested to be a tool with the potential to accelerate orthodontic movement, albeit with little change compared to the control side. The surgical approach to accelerating orthodontic tooth movement is currently more effective but is more invasive and expensive. Therefore, radiofrequency could be considered a useful, non-invasive, and comfortable tool to improve the speed of orthodontic treatment. This results in better patient compliance and fewer side effects, such as caries, gum recession, and root resorption. Future prospects are to evaluate on a number of patients sufficient to generate a proper statistical analysis, the consequent effects of the application of diathermy technology on the acceleration of tooth movement. This could, in the event of success, be a valuable tool to assist orthodontic treatments.

## Data Availability

All experimental data to support the findings of this study are available by contacting the corresponding author.

## Abbreviations

CAP	DiathermyCapacitive mode
EGF	Epidermal Growth Factor
HFA	High Frequency Acceleration
MCSF	Macrophage colony-stimulating factor
NITI	Nickel–Titanium
OPG	Orthopantomogram
PDL	Periodontal ligament
PGE	Prostaglandin E
PTH	Parathyroid hormone
RANK-L	Receptor activator of nuclear factor kappa-Β ligand
RES	Diathermy Resistive mode
TECAR	Capacitive and Resistive Energy Transfer
